# Trimetallic Nanoparticles: Greener Synthesis and Their Applications

**DOI:** 10.3390/nano10091784

**Published:** 2020-09-09

**Authors:** Mahmoud Nasrollahzadeh, Mohaddeseh Sajjadi, Siavash Iravani, Rajender S. Varma

**Affiliations:** 1 Department of Chemistry, Faculty of Science, University of Qom, Qom 37185-359, Iran; mhd.sajjadi@gmail.com; 2 Faculty of Pharmacy and Pharmaceutical Sciences, Isfahan University of Medical Sciences, Isfahan 81746-73461, Iran; 3 Regional Centre of Advanced Technologies and Materials, Palacký University in Olomouc, Šlechtitelů 27, 783 71 Olomouc, Czech Republic

**Keywords:** trimetallic nanoparticles, green chemistry, sustainable synthesis, eco-friendly methods, catalytic activities, nanoparticle synthesis

## Abstract

Nanoparticles (NPs) and multifunctional nano-sized materials have significant applications in diverse fields, namely catalysis, sensors, optics, solar energy conversion, cancer therapy/diagnosis, and bioimaging. Trimetallic NPs have found unique catalytic, active food packaging, biomedical, antimicrobial, and sensing applications; they preserve an ever-superior level of catalytic activities and selectivity compared to monometallic and bimetallic nanomaterials. Due to these important applications, a variety of preparation routes, including hydrothermal, microemulsion, selective catalytic reduction, co-precipitation, and microwave-assisted methodologies have been reported for the syntheses of these nanomaterials. As the fabrication of nanomaterials using physicochemical methods often have hazardous and toxic impacts on the environment, there is a vital need to design innovative and well-organized eco-friendly, sustainable, and greener synthetic protocols for their assembly, by applying safer, renewable, and inexpensive materials. In this review, noteworthy recent advancements relating to the applications of trimetallic NPs and nanocomposites comprising these NPs are underscored as well as their eco-friendly and sustainable synthetic preparative options.

## 1. Introduction

Nanoscience has the potential to make a healthy, safe, and valuable living environment where the industrial chemicals and toxic contaminants can be remediated by using relatively greener nanotechnologies [1,2]. Developing diverse nanomaterials possessing extraordinary and unique properties is definitely a pioneering way to resolve the challenges/problems for renewable energies, human health, effective drug delivery, and sophisticated medical treatments, but this area needs extensive explorations. Nano-scaled materials and nanoparticles (NPs) (especially metallic NPs) encompass novel and exceptional physicochemical and biological features [3,4,5,6,7]. Over the decades, they have shown broad applications in diverse fields ranging from catalysis, electronics, optoelectronics, bioengineering, sensing, tunable resonant devices, fuel cells, and information/energy storage, to nanomedicine [3,6,8,9,10,11,12,13,14]. Literature analysis reveals that the utilization of nanoscience and nanotechnology in assorted disciplines, namely chemistry, biology, engineering, and physics has led to the development and construction of advanced, efficient, and novel products with better-quality features, especially finding biomedical usability with a decrease in the adverse environmental/ecological impacts [14,15,16,17,18,19]. For instance, magnetic NPs and nanocomposites are of higher catalytic efficiency, antibacterial activity, easier to handle, and of lower operational costs; they are also endowed with the added benefits of recyclability, reusability, processing, sustainability, and targeting abilities [19,20,21,22].

According to the number of metals/metal oxides involved, metal NPs can be divided into single metals (monometallic) and bi- or trimetallic NPs. Among these materials, bi- and trimetallic NPs have garnered attention recently owing to their extraordinary properties, strong characteristics, and novel applications [23,24,25]. Compared to monometallic NPs, transition bi- and trimetallic NPs display higher catalytic selectivity/activity and excellent efficiency in numerous applications; they can be applied as heterogeneous nanocatalysts in various organic transformations [26,27]. Nanoscientists and researchers endeavored to fine-tune the size and/or morphology of these multimetallic NPs as heterodimer, core/shell, and alloys towards further catalytic utilizations [27,28,29,30]. Trimetallic NPs have recently received additional research awareness due to the innovative physicochemical characteristics (e.g., electronic, optical, catalytic, and magnetic) generated from the synergistic effects of their monometallic counterparts, especially in catalytic systems [31].

There are several synthetic approaches to prepare multimetallic NPs, namely electrochemical synthesis, chemical, and hydrothermal synthesis, coprecipitation, microemulsion, laser ablation, sol-gel, lithography, gas-phase condensation, microwave (MW) irradiation, mechanical alloying, and solvothermal technologies [32,33,34,35,36]. The literature reveals the preparation of trimetallic NPs as Sn-Zn-Cu [37], Pt_60_Ni_3_Cu_37_ [38], Au-Pt-Pd [39], and Au-Pt-Ag [40], among others (summarized in Table 1). For instance, it has been reported that the triblock copolymer Pluronic F127 could facilitate the preparation of Au@Pd@Pt layered core-shell-structured NPs comprising a gold core, palladium inner layer, and outer shell of nanoporous platinum [41]. Additionally, Au@Pd@Pt NPs were also synthesized by deploying poly(vinylpyrrolidone) (PVP) rather than Pluronic F127; PVP can be applied as a structure-directing and capping agent employed for fabricating varied metallic nano entities [42]. Transmission electron microscopy (TEM) images depict them as well-dispersed NPs with complex three-dimensional nanodendritic features. It appears that these feasible methods may be remarkably valuable options for commercial devices that incorporate Pt-based catalysts with significant catalytic active sites [42]. 

Some of these synthetic methods may have some drawbacks, namely the use of potentially hazardous reducing/capping agents (e.g., hydrazine and poly-*N*-vinylpyrrolidone) and toxic solvents. Additionally, the conventional physicochemical procedures entail time consuming, expensive/special equipment requiring high energy, pressure, and temperature, culminating in the formation of toxic byproducts/wastes and hazardous materials [6,43,44]. Therefore, the design of greener and sustainable synthetic routes is preferred since they are safe, environmentally salubrious, and often cost-effective [45,46,47,48,49]. Solvents and capping/stabilizing/reducing agents should be carefully considered based on the concepts and principles of green chemistry and be applied to such trimetallic NPs synthesis that entails the utilization of nontoxic reagents, capping/reducing agents, and safer solvents. There are several sustainable and facile greener synthetic routes based on biologically-derived chemicals or natural product extracts that are deployed as chemical reductants for the fabrication of nanostructures via the combination of these approaches with alternative activation means, including sonochemical [50], radiation-induced [51], electrochemical, and MW [52]. 

Green chemistry provokes the application of renewable materials to fabricate assorted nanomaterials with unique properties [65,66,67,68,69]; formation of trimetallic NPs via greener approaches deploying renewable resources is an ecologically beneficial undertaking [70,71,72]. In this review, state-of-the-art sustainable and greener syntheses of trimetallic NPs are discussed as well as their promising applications and associated important challenges.

## 2. Trimetallic NPs/Carbon Nanomaterial Composites with High Synergistic Effects

**Figure 1:**
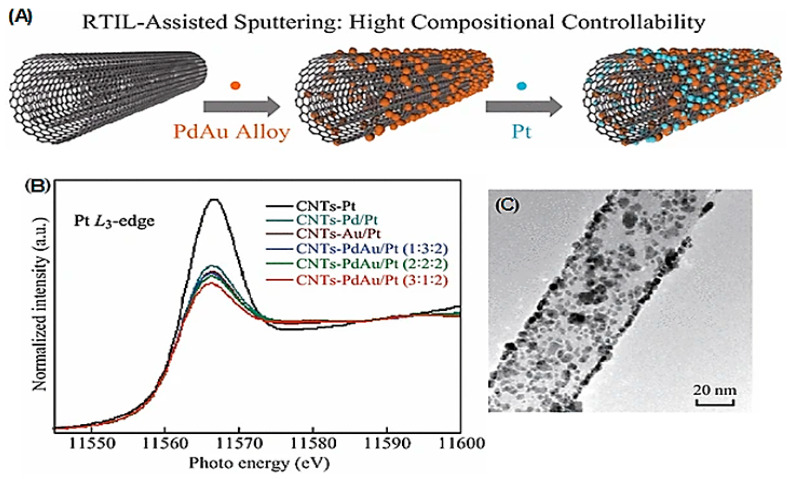
(**A**) The process of CNTs-PdAu/Pt NPs preparation, (**B**) Pt L_3_-edge X-ray absorption near edge structure (XANES) spectra of CNTs-supported PdAu/Pt trimetallic NPs, (**C**) TEM image of CNTs-PdAu/Pt NPs, Adapted from [76], (CC BY 4.0).

**Figure 2:**
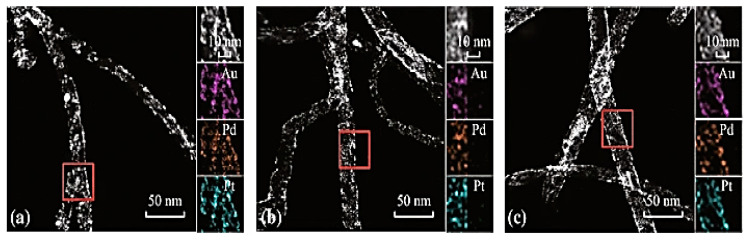
High-angle annular dark field scanning TEM (HAADF-STEM) images and regional elemental mapping of the Au, Pd, and Pt content of CNTs-PdAu/Pt trimetallic NPs with different Pd/Au/Pt ratios: (**a**) 1:3:2, (**b**) 2:2:2, and (**c**) 3:1:2 Adapted from [76], (CC BY 4.0).

The synergistic effects of metals, NPs, and/or their complexes have been evaluated [73,74]. In this regard, nanocomposites can provide several suitable applications comprising synergistic effects because of the manifestation of distinct components, mixed at nanoscale. Carbon-based nanomaterials with their remarkable optical, electrical, thermal, biocompatibility, and chemical properties can be utilized for various important applications [75]. Further, diverse biorenewable resources can be utilized for fabricating these carbon-based nanomaterials, including vegetable oils, isolated bio-chemicals, plant biomasses, and animal bio-products. In one study, the synergistic effects were detected in carbon nanotubes (CNTs)-PdAu/Pt trimetallic NPs (~3 nm) with excellent electrocatalytic performance and stability [76]. Tunable nanocomposites were designed by applying a straightforward physical approach encompassing room temperature ionic liquids (RTILs)-assisted sputtering deposition (Figure 1 and Figure 2) [76]. In another study, the immobilization of a series of electrocatalysts covering unitary, binary, and ternary metal boride oxides of Fe, Co, and Ni (with various permutations/combinations) was performed on polypyrrole/reduced graphene oxide (PPy/rGO) nanosheets. As a result, oxygen evolution reaction activities of the designed bimetallic electrocatalysts were higher than those of the monometallic electrocatalysts. Furthermore, the introduced trimetallic nanocomposite (FeCoNiBOx/PPy/rGO) exhibited optimal and significant oxygen evolution reaction activity, because of the synergistic effects of each component [77]. In this study, PPy/rGO provided a superior and precise surface area for immobilizing FeCoNiBOx, and significantly improved the oxygen evolution reaction activity of the electrocatalysts, by its good conductivity [77]. 

Interestingly, sustainable fabrication of graphene oxide (GO) has been facilitated via effective oxidation of graphite using sodium periodate (NaIO_4_) [78], where NaIO_4_ ensured oxidation, minimized defects on sp2 C-C plane and increased C/O atomic ratio of 5.146. The amino-functionalized reduced GO (NH_2_-rGO) and carboxylic surface modified Ag-Pt-Pd (COOH-AgPtPd) trimetallic NPs were fabricated to produce a label-free electrochemical sensing platform using the conventional immersive layer-by-layer (LBL) assembly. The superior synergetic effects between LBL-assembled NH_2_-rGO and COOH-AgPtPd (COOH-AgPtPd/NH_2_-rGO) significantly improved the electron transfer rate of the electrode as well as electrocatalytic efficiency. The ensuing COOH-AgPtPd/NH_2_-rGO nanocomposite-based sensing material was deployed for the non-enzymatic electrochemical detection of H_2_O_2_, with a low detection limit of 0.2 nM. Additionally, it was utilized as a sensing platform for the label-free accurate electrochemical detection of prostate-specific antigen (PSA) over a large linear range of 4 fg mL^−1^ to 300 ng mL^−1^, with an ultra-low LOD of 4 fg mL^−1^ [78]. In the following sections, we have introduced some other important examples of the designed nanocomposites adorned with trimetallic NPs as well as their suitable applications. 

## 3. Greener and Sustainable Synthesis of Trimetallic NPs

### 3.1. Bio-Based Synthesis

**Figure 3:**
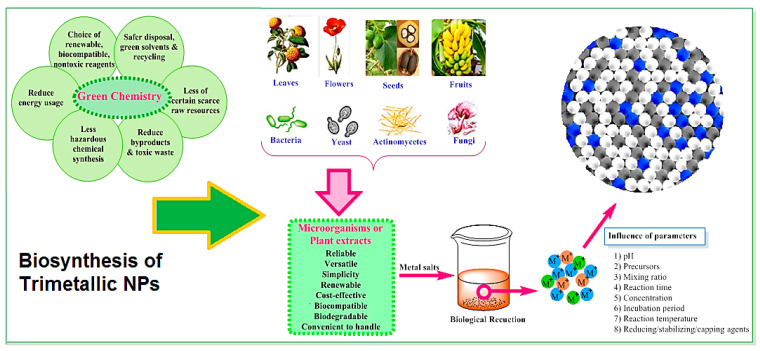
Bio-based synthesis of trimetallic NPs and their important advantages and parameters.

Generally, biologically-derived chemicals or natural product extracts obtained from bio-renewable sources with their promising reducing, stabilizing and capping potentials can be deployed for the greener production of mono- and multi-metallic NPs (Figure 3) [6,79,80,81,82,83]. However, there are still some important challenging issues persist, including the exact mechanisms for NP formation, limitations to scale up and the reproducibility of procedures [52]. Plant-mediated systems for the synthesis of mono/bi/trimetallic NPs, tubes, wires, rods, and alloys exploit the chemical constituents and ingredients of various plant systems, such as flavonoids, phenolic acid, glycosides, terpenoids, enzymes, proteins, vitamins, carbohydrates, alkaloids, and antioxidants (as capping, stabilizing, and reducing agents). The earliest example of using plant-based systems for the biosynthesis of mixed metal NPs dates back to 2007 by Haverkamp et al. [84]. They have fabricated Au-Ag-Cu alloy NPs (~5–50 nm) using *Brassica juncea* seeds via an in vivo approach.

Au/Pt/Ag trimetallic NPs have been produced via a facile, and environmentally friendly method by applying aqueous extract of *Lamii albi flos* [85]. Additionally, Au-ZnO-Ag trimetallic NPs (~20 nm) have been bio-prepared using the extract of *Meliloti officinalis* [86]. In another study, a reliable procedure was reported for the biosynthesis of Cu/Cr/Ni trimetallic oxide nanomaterials by applying *Froriepia subpinnata* and *Eryngium campestre* (aqueous leaf extracts) under mild temperature conditions [87]; these NPs exhibited superb antibacterial activities versus *E. coli* and *Staphylococcus aureus.* The optimized conditions for *E. campestre* leaf extract were 3 min reaction time, at 34 °C and metal salt-to leaf extract ratio of 2.38 with 73% yield. The corresponding value for *F. subpinnata* leaf extract was 3 min at 40 °C, and the ratio of metal salt to leaf extract was 1.07; 72% of the conversion of salt into trimetallic oxide NPs was evaluated by modeling software [87].

Alloy-like Ag-Au-Pd trimetallic NPs (~8–11 nm) have been green-fabricated in 10 min under ambient conditions by using extracts from *Aegle marmelos* (leaf extract) and *Syzygium aromaticum* buds, by altering the composition of phytochemicals [88]. These NPs exhibited promising catalytic activities for the glucose oxidation procedure, and demonstrated efficient antibacterial activities against Gram-negative bacteria, such as *E. coli*; high catalytic activity was presumably because of electronic charge transfer influence from the neighboring elements in trimetallic NPs which performed an important function [88]. 

### 3.2. Microwave (MW)-Assisted Synthesis

Compared with conventional synthetic approaches, MW-assisted synthesis have some important advantages, including rapid/uniform heating, energy savings, no selective heating of the surface, remarkable yield in shorter synthesis time, high purity of synthesized NPs and small narrow particle size distribution [89]. In one investigation, the formation, characterization, and synergistic antimicrobial activity of Au/Pt/Ag trimetallic NP-based nano-fluids have been reported [40]; these trimetallic nano-fluids were fabricated via a greener MW-mediated successive chemical reduction technique. Additionally, the MW irradiation method facilitated the fabrication of trimetallic nanocomposites and nanotubes. As a result, high-resolution scanning electron microscopy (HRSEM) images indicated that these particles were close to nanocomposite (Ag-Pt) and nanowire (Au) structures (length = 792 nm and width = about 99 and 51 nm) [90]. Additionally, HRSEM images of the fabricated Au-Pd-Pt trimetallic NPs revealed them to be of uniform nanotube shapes, length, and width of the nanotube being ~141 nm and 152 nm, respectively [90]. In another study, the authors reported on eco-friendly production of a Au/Pt/Ag trimetallic nanocomposite (~20–51.3 nm) by trisodium citrate-assisted reduction of ions under MW heating conditions [91]; the fabricated nanocomposites comprised gold nanowires and silver/platinum bimetallic NPs. The surface-enhanced Raman scattering (SERS), accomplished via the addition of 7-azaindole to trimetallic nanocomposites, and higher Pt content found on the surface [91]. It appears that the fusion of these three NPs into a single object would help improve the consistent localization of surface plasmon absorption [92]. 

The main advantageous feature of the trimetallic NPs is that there is suitable charge separation, as demonstrated in the heterostructure of matter, thus avoiding the charge recombination and rendering them as a promising alternative for photodegradation of organic contaminants. Additionally, these nanomaterials showed suitable adsorption ability and, consequently, they can be applied for the purification of wastewater laden with pharmaceutical wastes, especially antibiotics [93]. In one investigation, MW-assisted synthesis of La/Cu/Zr trimetallic NPs was accomplished and the ensuing nano-scaled material was deployed as a suitable nanophotocatalyst for removing the antibiotic, ampicillin, from an aqueous medium; the feasibility of ampicillin adsorbed on La/Cu/Zr trimetallic NPs demonstrated that the adsorption was accompanied with spur-of-the-moment photodegradation attaining 86% degradation [93]. 

The algal biochar reinforced trimetallic nanocomposites (~30–80 nm) were prepared by using greener MW synthesis method [94], and the ensuing nanocomposites were employed as adsorbent/photocatalyst for the exclusion of malachite green. These nanocomposites had remarkable adsorption/photocatalytic potentials for the remediation of malachite green (~94%) within 4 h [94]. In another study, the improved catalytic activity was exhibited by nanocomposites comprising GO supported monodispersed Pd, Ru, and Ni NPs (~3.78 ± 0.43 nm) [95]; these NPs adorned on GO were fabricated using the MW technique and they showed significant catalytic performance and stability for the dehydrocoupling of dimethylamine-borane with remarkable turnover frequency value (737.05 1 h^−1^) and activation energy (55.47 kJ mol^−1^) [95]. 

### 3.3. Ultrasonic-Assisted Synthesis

**Figure 4:**
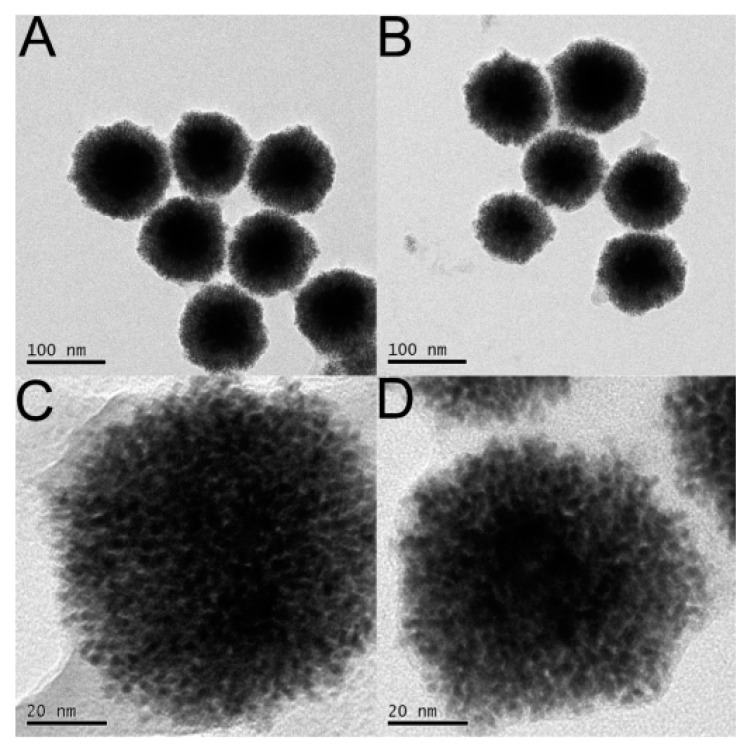
Trimetallic Pt-Au-Ru NPs constructed with the promising intermetallic phase for improving the fuel cells oxidation reaction (**A**–**D**). Reproduced from [54], with permission from Elsevier, 2018.

Sonochemical synthesis approach has been applied for facile fabrication of the trimetallic Pt-Au-Ru nanostructures with remarkably porous structures encompassing perpendicular pore channels (Figure 4) [54]. The prepared trimetallic nanocrystals with the augmented atomic ratio demonstrated superb electrocatalytic operations for oxidation of formic acid (the mass and specific activities are 1044.1 mA mg^−1^ and 14.5 mA cm^−2^, accordingly), 4.1 and 2.2 times more than attained from commercial Pt/C. It should be noticed that electrocatalytic activity and resiliency are important criteria for the electrocatalyst evaluations. It was reported that an electrocatalyst with improved durability showed considerable long duration, which is vital for industrial applications [54]. 

### 3.4. Laser Ablation Synthesis 

**Figure 5:**
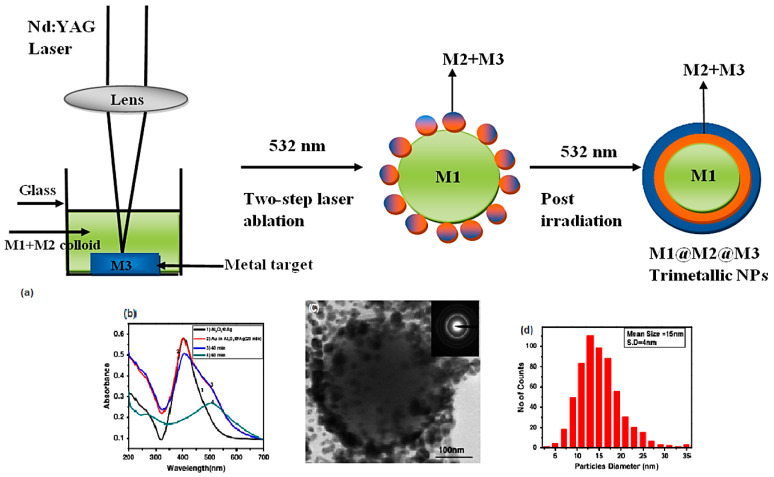
(**a**) The fabrication process of trimetallic nanoarchitectures by applying multiple-step laser ablation technique. (**b**) UV–VIS spectra, (**c**) TEM image, and (**d**) size distribution of Al_2_O_3_@AgAu NPs in deionized water. Reproduced from [100], with permission from Springer Nature, 2014.

Laser ablation synthesis in liquid solution is a green method for the synthesis of micro- and nanostructure directly from bulk materials, and is considered a green technique for producing trimetallic NPs without using any surfactant or addition of chemicals [96]. This method is environmentally friendly and cost effective, and can produce high-purity nanomaterials with no further contaminations although controlling the size distribution, agglomeration, and crystal structure are difficult [97]. Indeed, the precise quantitative analyses of the effects of pressure, temperature, and concentration of the ablated materials and concentration of the species in solution are vital for controlling the structure/phase of the final products [98]. In one study, trimetallic (Al_2_O_3_@AgAu) NPs were synthesized using nanosecond pulse laser ablation in deionized water [99]. Two-step laser ablation methodology was approved for the fabrication of these trimetallic NPs; a silver or gold target was ablated in colloidal solution of γ-alumina NPs synthesized by pulse laser ablation. These NPs showed high catalytic performance and were utilized for the catalytic reduction of 4-nitrophenol to 4-aminophenol. It was revealed that the catalytic efficiency of trimetallic NPs was significantly improved as they completed the catalytic reaction in only five seconds [99]. In another study, Al@Al_2_O_3_@Ag@Au and Al@Al_2_O_3_@AgAu triple-layered core-shell or alloy nanostructures were synthesized by a two-step laser ablation technique [100]. The preparative method included temporal separations of Al, Ag, and Au deposition for step-by-step generation of triple-layered core-shell structure. To produce Al@Ag NPs, silver was ablated for about 40 min in aluminum NP colloidal solution; aluminum get oxidized simply in water to generate alumina, the prepared structure thus possessed a core-shell Al@Al_2_O_3_ configuration. These Al@Al_2_O_3_ particles served as seeds for the incoming energetic silver particles to produce multilayered Al@Al_2_O_3_@Ag NPs. Then, the silver target was substituted by gold, and the ablation was performed to generate Al@Al_2_O_3_@Ag@Au core-shells or Al@Al_2_O_3_@AgAu alloy (Figure 5) [100]. 

### 3.5. Sustainable Synthesis Using Oleo Polyol Reagents

The utilization and improvement of novel and greener preparation protocol for the nanostructures preparation with controlled size and/or shape by sustainable reagents has been exemplified by oleo polyol and related polymers in recently [101], wherein novel Sr_0.3_Mg_0.7_Fe_2_O_4_ (SMF) trimetallic nanocubes were prepared via a solvent-less, cost effective oleo polyol reagents; the ensuing mesoporous trimetallic nanocubes exhibited excellent activities for wastewater treatment, and the removal of organic pollutants (Congo red) [102]. Three models (Freundlich, Temkin, and Langmuir) were utilized to study the adsorption isotherm and it was found that the magnetic SMF nanocubes with a large surface area (21.85 m^2^g^−1^) was able to efficiently adsorb Congo red (>90% within 30 min) [102].

### 3.6. Chitosan

**Figure 6:**
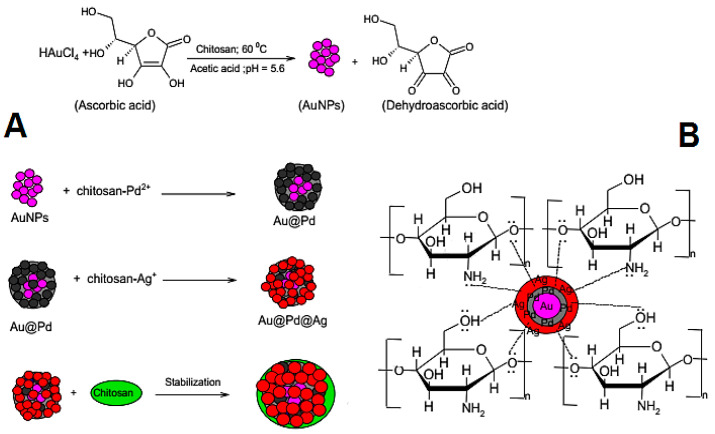
Schematic representation of seedless formation of Au-Pd-Ag NPs (**A**) and their stabilization using chitosan as capping agent (**B**). Reproduced from [103], with permission from Elsevier, 2020.

Chitosan is a naturally-occurring polysaccharide polymer with high biodegradability, significant biocompatibility, and nontoxicity which can be employed for synthesizing metallic NPs. For instance, chitosan was employed as a capping agent for fabrication of trimetallic Au-Pd-Ag with good stability; chitosan-Pd^2+^ and chitosan-Ag^+^ complexes were reduced on the surface of gold NPs (Figure 6) [103]. The NPs showed remarkable potentials as adsorbent for removing acid orange 7 from wastewater with maximum adsorption efficiency (~71.42 mg g^−1^); the maximum adsorption (90%) was detected under acidic pH (= 6.0) [103].

### 3.7. Glycine

Glycine was employed as a size-controlling agent for the surfactant-free one-pot production of ultrasmall homogeneous trimetallic Pt-Ni-Cu NPs (~2–5 nm) [104]. In this study, the synthetic significance of the water existing in the latter solvent system in promoting metal reduction was demonstrated, resulting in the production of a starting nanomaterial of about 5 nm. Additionally, the application of glycine can help stabilize the prepared ultra-small NPs with a suitable level of tenability and can be employed to control the size and morphology of the alloyed nanomaterials. Subsequently, compared with the commercially-available platinum black, the prepared NP showed significantly improved quality activity for the electrooxidation of ethanol [104].

## 4. Applications of Trimetallic NPs

### 4.1. Catalytic Applications 

**Figure 7:**
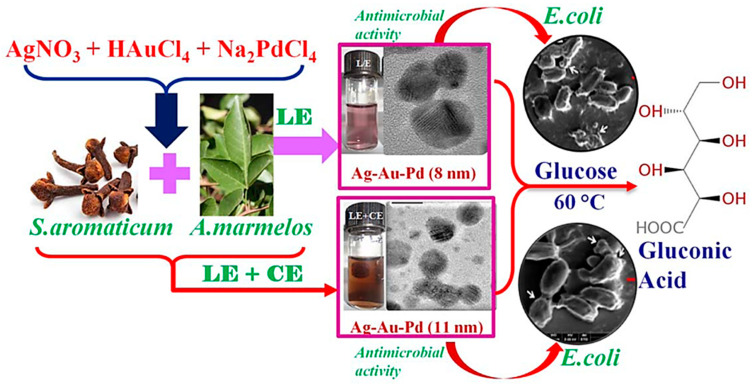
Schematic illustration of the fabrication of trimetallic NPs and their catalytic activities in the glucose oxidation and/or antimicrobial applications. Reproduced from [88], with permission from American Chemical Society, 2015.

Trimetallic NPs have recently garnered more attention as promising nanocatalysts in oxidation, degradation, and reduction reactions. In one study, alloyed metal NPs displayed excellent structural and physical features as compared to their bulk counterparts, e.g., improved solid solubility with reduced particle size [105]. Additionally, the green-fabricated and well-dispersed Ag-Au-Pd alloy NPs were obtained in a simple one-step protocol using the plant extract of *S. aromaticum* bud (CE) and *A. marmelos* leaf extracts (LE) as stabilizing/bioreducing agents and *Acacia auriculiformis* as a natural surfactant (Figure 7) [88]. The catalytic performance of Ag-Au-Pd trimetallic NPs achieved from LE (with a 5.26:2.16:1.0 atomic ratio) and LE+CE (with a 11.36:13.14:1.0 atomic ratio) were tested as a nanocatalyst for glucose oxidation process to afford gluconic acid under gentle circumstances without any external aeration. Further, the as-prepared trimetallic NPs revealed efficient antibacterial activities against *Escherichia coli* as a model Gram-negative bacterium [88].

A facile one-pot approach has been reported for the biogenic formation of trimetallic Au-Ag-Sr alloy NPs deploying *Coriandrum sativum* root extracts in an aqueous system without employing any external reductants/surfactants [106]. The trimetallic alloy NPs (spherical, ~70 nm) were utilized as an effective and recyclable nanocatalyst for the sensing of gases like methanol, ethanol, ammonia, and acetone. In another study, Au/CuO/ZnO trimetallic NPs were produced via a green method by applying aqueous extract of *Vitex agnus-castus* where these trimetallic NPs (spherical and agglomerated, 5–25 nm) exhibited potential for efficient catalytic treatment of wastewater, and the degradation of organic dyes such as crystal violet and methylene blue [107]. Generally, numerous bioactive substances of *V. agnus-castus* L., such as lipophilic flavonoids (chrysosplenol D and casticin), hydroxylated flavonoids, iridoid glycosides (agnuside and aucubin), were responsible for the formation of trimetallic-based NPs [107]. Additionally, trimetallic porous Au-Pd-Ru NPs exhibited remarkable catalytic activities for the reduction of *p*-nitrophenol, which can be employed for eliminating color from wastewater through the catalytic degradation of azo dyes. In addition, the amine produced can be removed from wastewater by adsorption on industrial solid waste dolomite [59].

**Figure 8:**
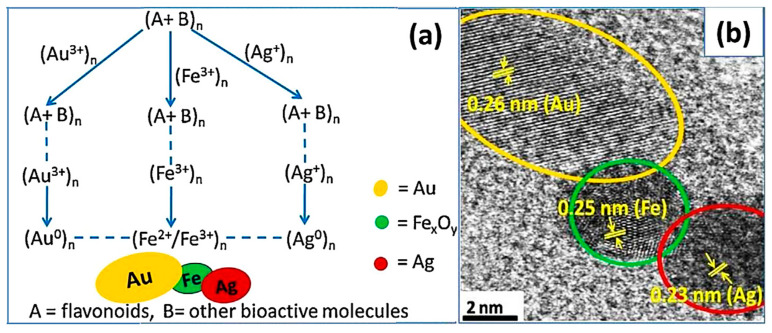
(**a**) Role of *A. membranaceus* for the Au-Fe-Ag NPs formation. (**b**) High-resolution TEM (HRTEM) image of Au-Fe-Ag NPs with D-spacing. Reproduced from [108], with permission from Elsevier, 2015.

**Scheme 1:**
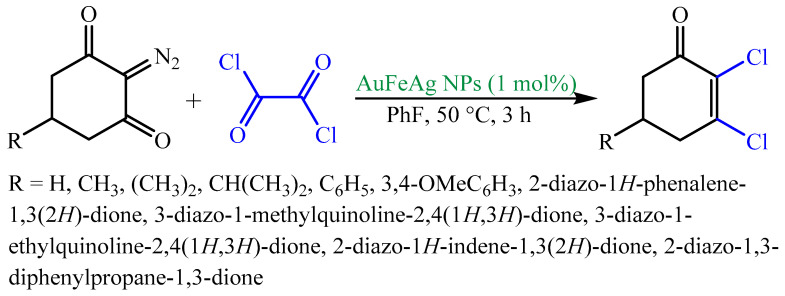
Au-Fe-Ag NPs-catalyzed efficient fabrication of various α,β- and β,β-dichloroenones.

Stable trimetallic Au-Fe-Ag NPs were produced in aqueous root extract of *Astragalus membranaceus*, which served as bioreducing and capping/stabilizing agents [108]. The hydroxyl and/or carbonyl groups of bioactive molecules from *A. membranaceus* plant, such as flavonoids, isoflavonoids, saponins, polysaccharides, and sterols, may be responsible for the reduction of Ag^+^, Au^3+^, and Fe^3+^ to Ag^0^, Au^0^, and Fe^2+^/Fe^3+^ NPs (Figure 8). The ensuing trimetallic hybrid NPs were applied as a magnetically retrievable green catalyst for the preparation of α,β- or β,β-dichloroenones from oxalyl chloride and diazodicarbonyl compounds (Scheme 1) [108].

**Figure 9:**
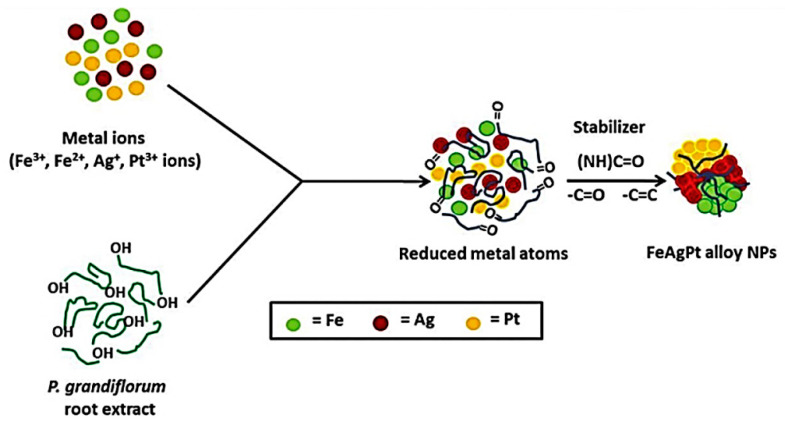
Schematic depiction of the role of *P. grandiflorum* in the Fe-Ag-Pt alloy NP formation. Reproduced from [27], with permission from Elsevier, 2017.

**Figure 10:**
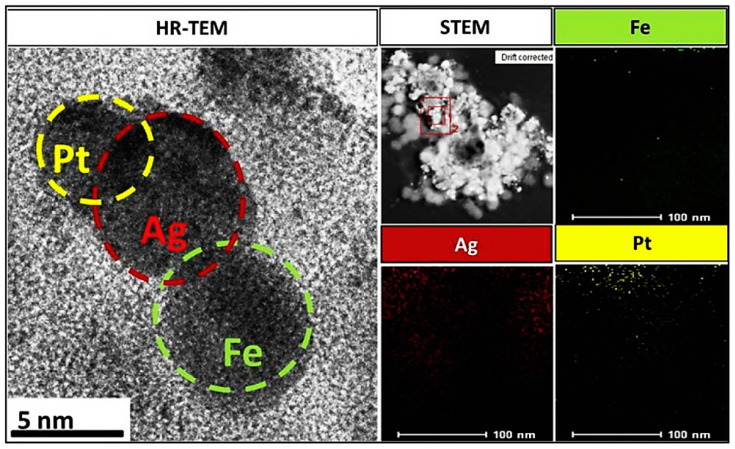
HRTEM images and HAADF-STEM images of Fe-Ag-Pt NPs and the elemental mapping of Pt, Fe, and Ag NPs. Reproduced from [27], with permission from Elsevier, 2017.

Yet another attempt for the utilization of trimetallic NPs in water treatment, and novel assembly of biogenic Fe-Ag-Pt alloy NPs have been made via a ultra-sonication procedure utilizing the root extract of *Pulicaria glutinosa* as stabilizer and bio-reducing agents (Figure 9) [27]. The ensuing trimetallic Fe-Ag-Pt alloy NPs (spherical, ~5 nm) can be deployed as a nanocatalyst for the remediation/reduction of highly toxic pollutants, namely Rhodamine B and 4-nitrophenol, using NaBH_4_ at ambient temperature from an aqueous solution (Figure 10) [27].

The bimetallic NPs have a tendency to undergo agglomeration during their repetitive application in catalysis [109,110]. In this context, a facile, and seedless, wet-chemical approach was utilized for the large-scale preparation palladium decorated gold/palladium core-shell nanocrystals (AuPd@Pd) by using HEPES (4-(2-hydroxyethyl)-1-piperazineethanesulfonic acid) buffer solution [111], served as shape-directing and reducing agents [112]. Through a simple ultrasonic treatment, the subsequent nanocrystals were uniformly loaded on the graphene nanosheets (G-AuPd@Pd), and tested as a green electrocatalyst (uniform spherical, 24.4 nm) for oxygen reduction and methanol oxidation reactions in an alkaline media. It was uncovered that the G-AuPd@Pd had improved catalytic activity, better stability, and high tolerance for these reactions, compared to the G-Pd (graphene supported Pd dendrites) or commercial Pd black catalysts [112]. In another study, a carbon cage-encapsulated Fe-Ni-Mo compound was synthesized and applied to electrocatalyze hydrogen evolution reaction [113]. This non-noble metal catalyst (with improved durability) demonstrated a significant electrocatalytic performance in both acidic (246 mV, 10 mA cm^−2^) and alkaline (199 mV, 10 mA cm^−2^) solutions. The advantage of this study was to find a promising approach to reduce the cost of the catalyst for the hydrogen evolution reaction by replacement of noble- metal catalysts by using efficient and durable non-noble metal catalysts [113]. Additionally, a facile and scalable strategy was designed for the production of *N*-doped bamboo-like carbon nanotubes decorated with trimetallic NPs to be employed for total water decomposition in alkaline electrolyte [114]. The coupling effect between various metals and the close contact with the carbon matrix give the resulting nanomaterial program excellent catalytic activity and stability, thereby promoting the hydrogen decomposition reaction and oxygen decomposition reaction and water-splitting in alkaline electrolyte. The prepared Fe-Ni-Cr/NC (with good durability) showed remarkable catalytic activity for hydrogen evolution reaction relative to Fe-Cr/NC, Ni-Cr/NC and Fe-Ni/NC [114]. 

Trimetallic NPs can be applied as electrocatalysts for glycerol’s selective oxidation [56], as exhibited by trimetallic Pt_x_Au_y_@Ag catalysts in alkaline media relative to acidic solutions; these catalysts demonstrated remarkable current density (0.67 mA cm^−2^), about 2.6 times higher than the Pt/C catalyst. Under alkaline conditions, the Pt_4_Au_6_@Ag had significant current density (3.1 mA cm^−2^), about 4.7 times higher than the Pt/C catalyst [56]. Oxidation products from glycerol oxidation over these catalysts, as evaluated by high-performance liquid chromatography (HPLC), comprised nine products, namely, glyceric acid, oxalate acid, tartronic acid, glyceraldehyde, glyoxylic acid, glycolic acid, lactic acid, formic acid, and dihydroxyacetone (DHA) [56].

Interestingly, the single-phase structured nanoalloy of Pt-Au-Co exhibited dehydrogenation of ammonia borane (NH_3_BH_3_) with Pt_76_Au_12_Co_12_ nano-alloy being the ideal one demonstrating room temperature high TOF value of 450 (mol H_2_ min^–1^ (mol metal)^−1^) [115]; kinetic analysis confirmed that the Pt_76_Au_12_Co_12_-triggered catalytic hydrolysis, was of first-order with an activation energy as low as 18.47 kJ mol^–1^. X-ray photoelectron spectroscopy (XPS) and intracranial pressure (ICP) analyses revealed that the altered electronic interaction played a critical function in the augmented catalytic performance of the Pt_76_Au_12_Co_12_ nano-alloy [115].

The preparation of graphene nanocomposites by engaging appropriate composite nanostructures that rendered an excellent synergetic effect between the constituent components and provided a good anchoring site for the insertion and/or stabilization of metal/metal oxide NPs is still a main challenge. While maintaining the exceptional features of each composite in the synthesis of good homogeneous nanocomposites, the layer-by-layer (LBL) deposition technique fulfils all these requirements [116,117,118]. One of the effective and key characteristics of LBL assembly for the fabrication of differently layered nanocomposites is the functionalization of nanomaterials; these nano-based biosensors could render promising nanoplatforms for the advancement of other biosensors [118].

**Figure 11:**
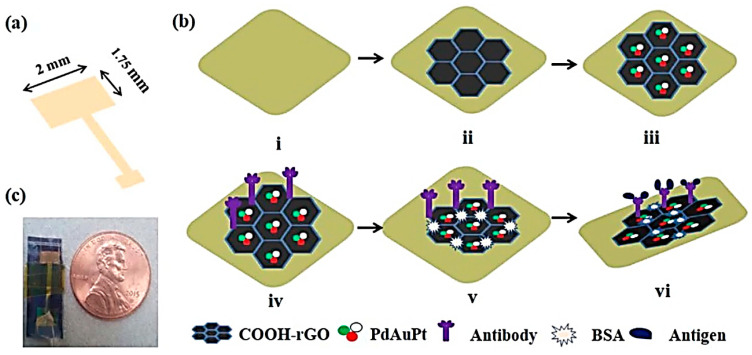
Schematic illustrations of the (**a**) sensing platform, (**b**) preparation sequence of trimetallic PdAuPt/COOH-rGO immuno-sensor for the CEA/PSA detection; photomicrograph of the (**c**) advanced immunosensors. Reproduced from [119], with permission from Elsevier, 2018.

Barman et al. [109] have developed a trimetallic electrocatalytic surface by electrodeposition of Pd-Au-Pt NPs on carboxyl terminated reduced GO (–COOH-rGO) under optimum processing conditions. It was applied as electrochemical immunosensor nanoplatforms for fast and sensitive detecting PSA and carcinoembryonic antigen (CEA) biomarkers (Figure 11). After treatment, the anti-PSA and anti-CEA can be immobilized separately on two diverse PSA and CEA antibody-conjugated PdAuPt/COOH-rGO platforms [119].

A recyclable nanocatalyst, Fe_3_O_4_@SiO_2_@Co-Zr-Sb, was employed in an efficient, facile, and environmentally friendly method for the direct reduction of nitroaromatics to the corresponding azoaromatics [64]. The chemical selective hydrogenation of nitrobenzene was carried out in refluxing water to obtain ethoxybenzene (approximately 2–10 min with high yield). In addition, the catalyst was employed continuously four times with no significant activity failure. Some notable features comprise short reaction time, elevated yields, no harmful organic solvents, mild reaction conditions, with the utilization of recyclable nano-magnetic catalysts and deployment of water as a green solvent [64].

### 4.2. Biomedical Applications 

#### 4.2.1. Sensing Applications

Cu-Au-Pt trimetallic NPs with intense plasmonic absorption in the near-infrared fluorescence imaging (NIR-I) bio-window (~650–950 nm) and significant catalytic activity were fabricated for biosensing and theranostics applications, considering the potential combination of various enzyme-related reactions and different molecular recognition devices [120]. By harnessing the catalytic characteristics of Cu/Au/Pt NPs, a sensitive colorimetric sensing technique was developed for the precise evaluation of glucose. Additionally, an innovative theranostic probe was produced by these thiolated aptamer-coated trimetallic NPs, which could efficaciously achieve visual, sensitive, and selective analysis, and remarkable photothermal demise of target cancer cells [120]. In another study, the nanocomposite sensor (based on Au-Ag-Pd NPs) which was uniformly capped by electro-pretreated GO has been fabricated and applied for the simultaneous determination of ascorbic acid, dopamine, acetaminophen, and tryptophan. This nanocomposite electrode can be practically employed for the analysis/determination of these substances in real samples of human blood serum [121].

**Figure 12:**
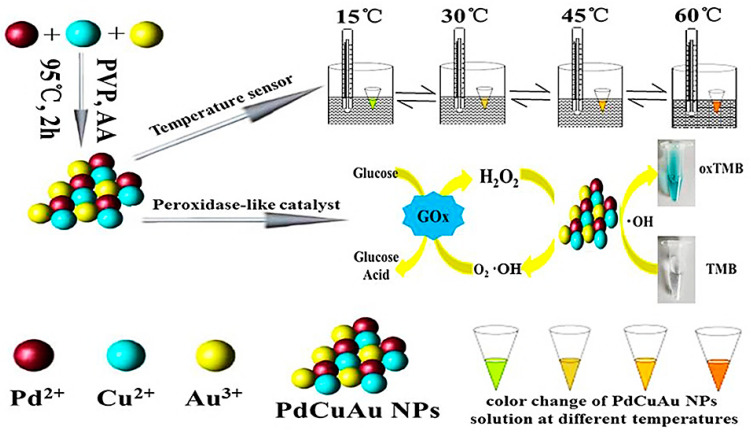
Pd-Cu-Au trimetallic NPs synthesized to be applied as temperature sensors and for colorimetric H_2_O_2_ detection as well as glucose with enzyme mimic. Reused from [122]. An Open Access Article, (CC BY 4.0).

Interestingly, Pd-Cu-Au trimetallic nanomaterials with good stability were synthesized by the simple one-pot method, and exhibited potential as temperature sensors for sensitive detection of changes in fluorescence intensity (in about 4–95 °C temperature range), because of their unique optical characteristic [122]. Additionally, the trimetallic NPs showed significant catalytic operation for peroxidase-like enzymes; they could catalyze 3,3’,5,5’-tetramethylbenzidine (TMB) promptly in the presence of H_2_O_2_ and oxidatively convert it to a visible blue product (oxTMB). Thus, these trimetallic NPs can be employed as colorimetric platform for detection of H_2_O_2_ (0.1–300 μM, LOD = 5 nM) and glucose (0.5–500 μM, LOD = 25 nM) (Figure 12) [122]. 

#### 4.2.2. Cancer Therapy and Diagnosis

**Figure 13:**
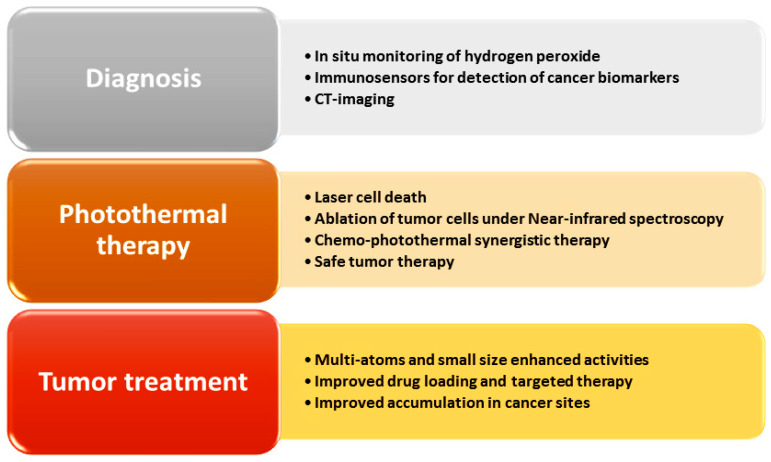
Anticancer potentials of trimetallic nanomaterials.

In addition to the available therapeutic options, cancers are still considered as foremost diseases causing enormous human mortality. Thus, the treatment and diagnosis of cancers in early stages are vital, and in this regard, trimetallic NPs show promising potentials and should be emphasized by researchers in the field (Figure 13). Additionally, developing sensitive, fast, and affordable electrochemical immunosensors for detecting cancer biomarkers (vital in primary diagnosis of cancers for better managements and treatment) is a challenging issue [123].

##### Electrochemical Sensors for Cancer Cells

**Figure 14:**
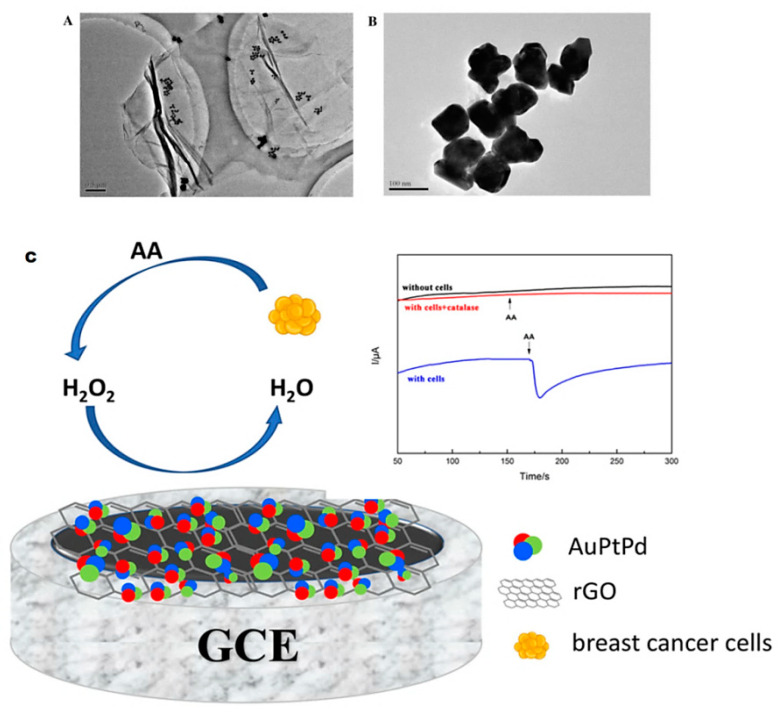
(**A**,**B**) TEM images of varying magnifications of AuPtPd/rGO. (**C**) AuPtPd/rGO-modified GCE employed for identifying H_2_O_2_ released from cells. Reproduced from [39], with permission from Elsevier, 2018.

Precisely applying a biomarker to identify H_2_O_2_ under physiological circumstances is crucial to find the signal transduction pathways, which are essential in early cancer diagnosis [39]. Interestingly, a sensor for the detection of H_2_O_2_ was prepared by deploying trimetallic Au-Pt-Pd nanocomposites decorated on reduced GO (rGO) nanosheets with the amendment of the rGO and by the physical adsorption of trimetallic NPs on a glassy carbon electrode (GCE) (Figure 14). This prepared electrode showed remarkable electrocatalytic activity for the hydrogen peroxide (H_2_O_2_) reduction comprising a low detection limit of 2 nM and a wider linear range from 0.005 μM to 6.5 mM, suitable selectivity and approved resilience. Additionally, the produced sensor is capable of monitoring H_2_O_2_ release from living cancer cells, and it can be employed for initial diagnosis of cancers [39]. Additionally, in situ detection of secreted H_2_O_2_ from live MCF-7 cancer cells using trimetallic hybrid nanoflower-adorned molybdenum disulfide (MoS_2_) nanosheet sensor was reported [124]. Consequently, the resulted composites showed remarkable catalytic activities for the electrochemical reduction of H_2_O_2_ because of the synergistic effects of the prepared nanoflowers and MoS_2_ nanosheets, which provided an in vitro ultrasensitive H_2_O_2_ monitoring. Further, the laminin glycoproteins immobilization of the prepared nanocomposites surfaces improved the biocompatibility for growth and cell adhesion that allowed in situ electrochemical detection of secreted H_2_O_2_ from live cells for applications in the field of clinical diagnosis, biosensors in cellular biology, and pathophysiology of cancers [124]; such sensors can be employed for early cancer diagnostics, cellular biology, and pathophysiology.

**Figure 15:**
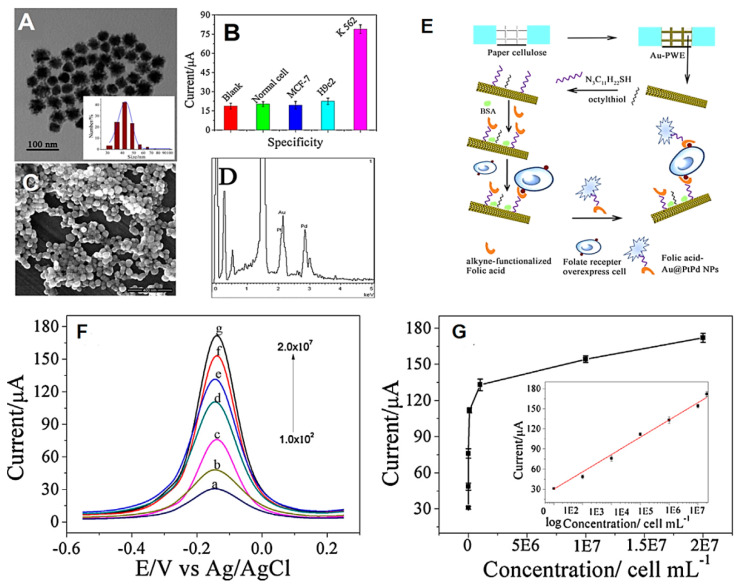
TEM image of dendritic Au@PtPd NPs (**A**), peak current response of the electrochemical sensor from the solution with various cell lines (**B**), SEM image of dendritic Au@PtPd NPs (**C**) and Energy-dispersive X-ray spectroscopy (EDS) image of dendritic Au@PtPd NPs (**D**). Schematic illustration of electrochemical sensor of cancer cells by deploying folic acid modified Au@PtPd NPs on paper device (**E**). The differential pulse voltammetry (DPV) curves of the electrochemical sensors toward various concentration of K-562 cell (**F**), calibration curve (inset, logarithm calibration curve) (Notes: pH 7.0PBS, 2 mM thionine and 10 mM H_2_O_2_ incubation for 20 min at room temperature) (**G**). Reproduced form [125], with permission from Elsevier, 2015.

Trimetallic dendritic Au@PdPt NPs could catalyze the H_2_O_2_ reduction with the assistance from thionine, the electron mediator, producing noticeable improvement in the electrochemical signals [125]. The production of a sandwiched sensor and its implementation in the form of a paper chip device for determining K-562 cells was achieved; the click chemistry was used for linking the folic acid with the surface of dendritic Au@PtPd NPs, thus facilitating the attachment of folic acid to the folate receptor (FR) of cell surface with significant attraction and assaying K-562 cells on folic acid-modified Au@PtPd NPs [125]. An origami electrochemical device adorned with a folic acid-modified Au-paper working electrode was prepared that could capture K-562 cells. The folic acid applied for capturing K-562 cells and attaching the fabricated dendritic NPs nanoprobe can accelerate the sensorial selectivity for detection of the cells. A superior analytical performance of this biosensor for cytosensing of K562 cells (detection linear range of about 1.0 × 10^2^ to 2.0 × 10^7^ cells mL^−1^, LOD = 31 cells mL^−1^) was demonstrated. The improved sensor response by applying these trimetallic NPs was detected instead of single Ag, Pt and Pd NPs (Figure 15). Thus, this sensor can be applied for point-of-care diagnostics with good sensitivity [125]. In another impressive study, an ultrasensitive sandwich-type electrochemical immunosensor was constructed for the quantitative detection of carcino-embryonic antigen [126] where trimetallic NiAuPt NPs were applied on graphene nanosheets as outstanding labels and *β*-cyclodextrin functionalized rGO nanosheets as platforms. These graphene nanosheets with remarkable specific surface area, high biocompatibility, and significant dispersibility were deployed for the efficient capture of the primary antibodies. These trimetallic nanocomposites were found suitable as labels for signal amplification, demonstrating improved electrocatalytic activity for reducing H_2_O_2_, much better than that the monometallic or bimetallic ones due to the inherent synergetic effects. Under the optimized conditions, a linear range from 0.001–100 ng mL^−1^ and LOD of 0.27 pg mL^−1^ were achieved for carcino-embryonic antigen. This immunosensor showed significant analytical performance for the measurement of carcino-embryonic antigen (in clinical diagnostics) with low LOD, wide detection range, high selectivity, good reproducibility, and stability [126]. 

**Figure 16:**
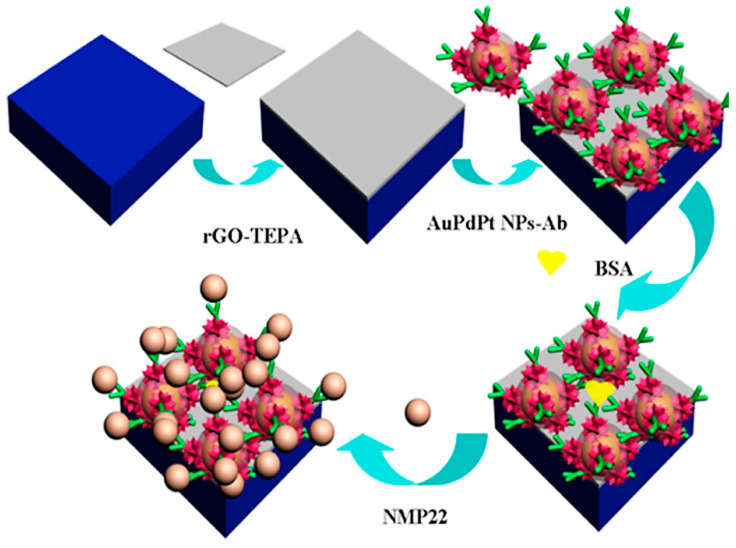
Schematic illustration of immunosensor for monitoring cancer biomarkers based on rGO-TEPA and trimetallic Au-Pd-Pt NPs. BSA: bovine serum albumin. Reproduced from [123], with permission from Elsevier, 2015.

The identification of bladder cancer biomarker-nuclear matrix protein 22 (NMP22) was accomplished by deploying a sensitive electrochemical immunosensor exploiting reduced GO-tetraethylene pentamine (rGO-TEPA) and trimetallic Au-Pd-Pt NPs. rGO-TEPA was employed for signal augmentation and immobilization of NPs due to its larger surface area and high conductivity. The operative system was produced for the anchoring of antibodies by applying trimetallic NPs, which retained the significant bioactivity and stability of antibodies [123]. Additionally, the trimetallic NPs did increase the electron transfer and improve the response of the signal, which were supported by synergistic influences of Au, Pd, and Pt. It was revealed that this immunosensor, prepared with a simple fabrication method, showed low detection limits of 0.01 U mL^−1^ and widespread linear range of 0.040–20 U mL^−1^. Additionally, it showed a short analysis duration of about two min for the detection of NMP22 with remarkable selectivity and stability for (Figure 16). This immunosensor can be employed for the sensitive, selective, and specific monitoring of cancer biomarkers and, thus, is suitable for cancer diagnostics [123]. 

#### 4.2.3. Antimicrobial Activities 

**Figure 17:**
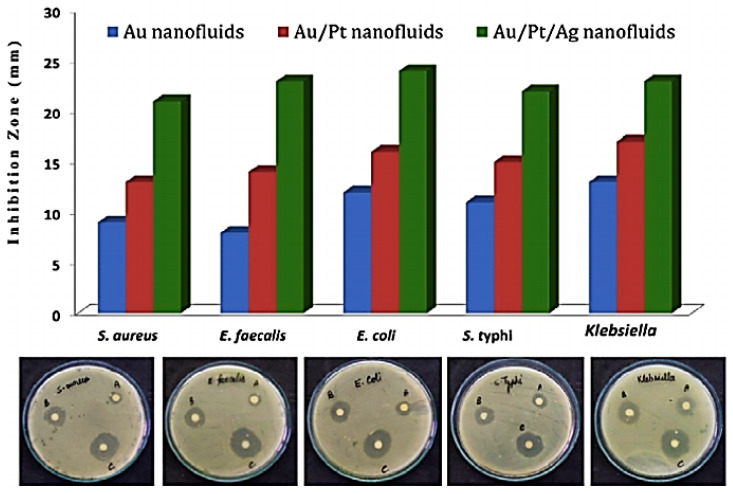
Antibacterial activities of the produced nanomaterials in terms of inhibition zone; Histograms provided comparative zone inhibition of the nanofluids against various bacteria; ‘A’, ‘B’ and ‘C’ in the agar plates correspond to Au, Au/Pt, and Au/Pt/Ag nano-fluid, respectively. Reproduced from [40], with permission from Elsevier, 2018.

Trimetallic NPs exhibited efficient and promising antimicrobial potentials than the mono and bi-metallic counterparts. As an example, CuO-NiO-ZnO mixed metal oxide NPs (~7 ± 2 nm) displayed good antibacterial activities and damaging effects against *E. coli* and *S. aureus* bacterial strains; the NPs attached to the bacterial cell wall and totally annihilated the cells [127]. In another study, the green-synthesized trimetallic Au-Pt-Ag NPs (~35–40 nm) exhibited potential for anti-biofilm and antimicrobial activities against *S. aureus*, *E. coli*, *Enterococcus faecium*, *Enterococcus faecalis*, and *Candida albicans* [85]. It was revealed that inhibition responses of trimetallic nanomaterials were higher than monometallic and bimetallic ones, as in the case of Au/Pt/Ag trimetallic nanomaterials synthesized by MW-assisted method (Figure 17). Some important mentioned mechanisms for antibacterial effects of these nanomaterials are the formation of reactive oxygen species (ROS) (as the main cause of cellular death), membrane modifications, and a decrease in ATP level and inhibition of tRNA binding to the ribosome [40]. 

Over the last few years, several efforts have been made for in situ generation of ZnO NPs [128,129], TiO_2_ NPs [129,130], and Ag NPs [131] onto cotton fabrics whose benefits include the reduced time for the finishing procedures, low-cost procedures, and prevention of environmental pollution. In this context, a facile, environmentally viable, economic, and versatile approach has recently been introduced with reduced-time operation for surface amendment of cotton fabrics to render them multifunctional in a single-step process [132]. This modification prompted the preparation of metallic/metal oxide NPs inside cotton fabrics so that they could be loaded with Ag NPs, Ag/ZnO bimetallic NPs, or Ag/ZnO/Cu trimetallic NPs. It was observed that the preparation and stabilization of these mono-, bi- and trimetallic NPs by using polymethylol compound (PMC-AZ, PMC-A, and PMC-AZC) and functionalized polyethyleneimine (FPEI-AZ, FPEI-A, and FPEI-AZC) in a bath comprising cotton fabrics induces the modified cotton fabrics with unprecedented properties, e.g., antibacterial, ultraviolet protection, and conductivity. As a result, the solution of three metal compounds can be changed from colorless to yellow and black green color by addition of FPEI or PMC polymers. According to the UV spectra, FPEI or PMC exhibited no specific peaks and the appearance of the absorption maximum at 410–415 nm confirmed the successful preparation of Ag NPs stabilized by FPEI and/or PMC chains.

### 4.3. Active Food Packaging 

Due to poor mechanical and barrier characteristics of biopolymers, their applications are restricted in food packaging. Therefore, the incorporation of innovative NPs into biopolymers may improve these characteristics [133]. Trimetallic NPs (like Au-Fe-Ag and Fe-Ag-Pt) can act as promising antimicrobial agents for food packaging applications [134]; however, limited data are available on the passage of NPs into human organs and also their toxicity analyses in this field of science [133]. Innovative synthesis of trimetallic NPs and their incorporation into biopolymers are expected to improve the shelf life of foods, including preserved food, vegetables, fruits, cooked meat, fish, processed foods, cakes, bread, fermented milks, and dairy foods. However, evaluations regarding the toxicity issues of such ensued nanocomposites used for food packaging should be considered systematically and critically before production, supply, and distribution of the product; the risk assessments and safety aspects of the complex NPs should be added to the priority for research [133].

### 4.4. Removal of Antibiotics and Organic Pollutants

MOFs, metal-organic frameworks, are an emerging and fascinating class of porous hybrid organic-inorganic materials prepared from metal ions or clusters and organic ligands linked by coordinating bonds, and have promising potential for sensing, gas storage, and catalysis [135]. The suitable compatibility of these hybrid materials with different metal ions can produce homogeneous multi-metallic spinels, with unique characteristics attributable to both the MOFs and the metal ions with promising functionalities [136]. In one investigation, magnetic NiCo/Fe_3_O_4_-MOF-74 was prepared by applying a precursor trimetallic NiCoFe-MOF-74. It exhibited significant stability, remarkable magnetism and active metal sites with respect to the original MOF. It showed suitable enrichment ability to remove antibiotics, like tetracycline (about 94.1% within 5 min, reusable five times without significant loss), which was more than NiCoFe-MOF-74 [137]. The open metal sites to generate a stable metal-ligand with the antibiotic molecules make it a suitable framework for prompt removal of antibiotics from water at a low cost and with high efficiency [137].

Fe-La-Zn@GO trimetallic nanocomposite (~17 mm) and Fe-La-Zn trimetallic NPs (~19 mm) have been employed as photocatalysts for removal of organic pollutants [138]. They were investigated for sunlight-induced phenylhydrazine photodegradation, and by applying Fe-La-Zn trimetallic NPs (optical band gap of about 2.73 eV) and Fe-La-Zn@GO trimetallic nanocomposite (band gap of about 2.30 eV), the reduction of 52 and 57.91% was obtained, accordingly [138]. The photocatalytic activity of the MW-synthesized La/Cu/Zr/carbon quantum dots trimetallic nanocomposite was explored for removing persistent pollutants, including ampicillin antibiotic and malachite green dye, up on visible light exposure [61]. Consequently, ampicillin antibiotic 96% and malachite green 86% have been destroyed in four hours of photo-irradiation via adsorption in dark followed by a photocatalysis and coupled adsorption/photocatalysis procedure [61]. The adsorption of antibiotics and dyes occurred on the trimetallic nanocomposite’s surface. The photocatalysis procedure involved in the production of electron-hole pairs on light irradiation of the trimetallic nanocomposite, which reacted with water molecules to generate super oxide and hydroxyl radicals causing contaminant (like dyes or antibiotics) mineralization [61].

## 5. Conclusions

In conclusion, the design of suitable sustainable and greener synthetic methods for producing trimetallic NPs with well-defined morphologies/compositions and desired activities is crucial. Further, the optimization of reaction parameters, including pH, time, stabilizing/capping agents, reducing agents, nature/concentration of precursors, and temperature is rather critical and need to be addressed. Trimetallic NPs show unique characteristics and display more reactivity than bi- and monometallic nanomaterials and, hence, can be employed as promising nanocatalysts, antimicrobials, and anticancer agents because of their enhanced electrochemical activity, better catalytic performance and higher stability; they are promising photocatalysts and adsorbents for the elimination of hazardous pollutants and contaminants. Trimetallic catalysts have significant potential as signal amplifying nanoprobes for ultrasensitive electrochemical sensing applications. 

The development of greener techniques using less hazardous and toxic solvents, fewer chemical reagents, precursors and catalysts, and involving fewer steps in the synthetic pathway have been the focus of researchers; scanty information is, however, available on the synthesis of trimetallic NPs via such greener routes. Forthcoming investigations should systematically address important challenging issues as well as innovative sustainable and greener methodologies for designing well-organized trimetallic NPs with capabilities to incorporate earth-abundant materials under eco-friendly conditions. Additionally, in view of deployment of trimetallic NPs for diverse catalytic, active food packaging, water treatment and biomedical applications, the following important future perspectives need to be addressed:

1. The biocompatibility, toxicity, and histopathological evaluations of the synthesized trimetallic NPs and nanocomposites should be systematically and analytically performed, especially for biomedical applications (in vitro/in vivo tests); these metallic nanomaterials may contribute to secondary pollution and, hence, these analyses are imperative.
2. The effects of optimization conditions, isolation/purification techniques, and related reactions factors on the behavior/performance of trimetallic NPs and their size and morphology need to be meticulously attended.
3. The cost-effectiveness and scale-up evaluations should be attended to compare these sustainable and greener synthetic methods with existing conventional physicochemical strategies.

## Figures and Tables

**Table 1:** Some important examples of the synthesized trimetallic NPs by various methods.

Trimetallic NPs	Method	Size	Morphology	Refs.
Au-Pd-Pt	Simultaneous reduction	1.7	Round	[53]
Pt-Au-Ru	Ultrasonic-assisted synthesis	77	Spherical	[54]
Au-Pt-Pd	Simultaneous reduction	80–100	Irregular	[39]
Au-Pt-Pd	Successive reduction	75–90	Dog-bones shaped	[55]
Au-Pt-Ag	MW irradiation	23	Dark nano-fluids	[40]
Pt-Au-Ag	Seed-mediated-growth	40–50	Spherical	[56]
Au-Pd-Pt	Seed-mediated-growth	55	Island cluster	[57]
Au-Pd-Ag	Seed-Mediated co-reduction	30	Polyhedral	[58]
Au-Pd-Ru	Seed-Mediated growth	110	Blackberry like	[59]
Ag-Au-Pt	Galvanic replacement	70	Porous nanocages	[60]
Sn-Zn-Cu	Chemical reduction	20	Core-shell structure	[37]
Pt-Ni-Cu	Hydrothermal method	40	Porous structure	[38]
La/Cu/Zr/carbon quantum dots	MW-assisted synthesis	30–100	Fibrous	[61]
Ag-Cu-Pt	Co-reduction method	32.89 ± 4.35	Core-shell structure	[62]
Fe-Ag-Pd	seed-less simultaneous chemical reduction	Capsules-like: 93 & nano-rod: 50	Capsules-like and nano-rod like	[63]
Co-Zr-Sb	Co-precipitation method	18–23	dark cores surrounded by the other shells	[64]
